# Metastatic Anaplastic Thyroid Carcinoma Presenting with Gastrointestinal Bleeding: A Case Report and Literature Review

**DOI:** 10.3390/reports9020185

**Published:** 2026-06-14

**Authors:** Hassan Al-Thani, Husham Abdelrahman, Maryam Al-Sulaiti, Abdelhakem Tabeb, Mahir Petkar, Noora Al-Thani, Ayman El-Menyar

**Affiliations:** 1 Department of Surgery, Trauma Surgery Section, Hamad General Hospital (HGH), Doha P.O. Box 3050, Qatar; halthani@hamad.qa (H.A.-T.); habdelrahman2@hamad.qa (H.A.); 2 Department of Surgery, Hamad General Hospital (HGH), Doha P.O. Box 3050, Qatar; malsulaiti4@hamad.qa (M.A.-S.); atabeb@hamad.qa (A.T.); 3 Anatomy Pathology, Laboratory Medicine and Pathology, Hamad General Hospital (HGH), Doha P.O. Box 3050, Qatar; mpetkar@hamad.qa; 4 Department of Medicine, Endocrinology, Hamad General Hospital (HGH), Doha P.O. Box 3050, Qatar; nalthani@hamad.qa; 5 Department of Surgery, Trauma & Vascular Surgery, Clinical Research, Hamad Medical Corporation, Doha P.O. Box 3050, Qatar; 6 Department of Clinical Medicine, Weill Cornell Medical College, Doha P.O. Box 24144, Qatar

**Keywords:** thyroid, anaplastic carcinoma, metastasis, surgery, gastric metastasis, endoscopy

## Abstract

**Background and Clinical Significance**: Thyroid cancer is increasing, particularly the differentiated type, with decreasing incidence of the anaplastic type. Anaplastic thyroid carcinoma (ATC) is a rare, aggressive, and often lethal form. It frequently presents with metastatic disease, regional and systemic, with common distant metastasis to the lung, bone, brain, and adrenal, and rarely to other places; **Case presentation**: A 74-year-old Arab male presented with symptomatic anemia and melena and was admitted for investigation of the cause. The patient was found to have a large retrosternal goiter and gastric tumor. CT scan showed a pedunculated, nonobstructive mass, suggestive of a GIST or leiomyoma. The neck mass presented with compressive symptoms. He underwent a combined neck and abdominal surgical resection based on a multidisciplinary team decision, as prior biopsies were not conclusive. The final pathology report identified similar tumors in the two specimens and suggested an anaplastic thyroid carcinoma as the primary tumor with metastasis to the stomach. Furthermore, the workup, including a PET scan 2 weeks post-surgery, revealed widespread metastases in the bone, lung, and liver, and the treatment was palliative. He was followed up in the outpatient clinic for 4 and a half months post-operatively. The patient developed sepsis and cardiopulmonary arrest and died; **Conclusions**: ATC can metastasize to many places in the body, including the stomach (as shown in our case), which can cause significant upper gastrointestinal bleeding and anemia. Metastatic ATC carries a poor prognosis; thus, physicians need to keep a high index of suspicion in approaching similar cases. A multidisciplinary approach for the management is of utmost importance for appropriate treatment. This disease’s pathology, behavior, and targeted new treatment modalities must be explored further.

## 1. Introduction and Clinical Significance

Thyroid cancer incidence has increased worldwide, mainly because of the differentiated papillary types. Anaplastic thyroid carcinoma (ATC) is a rare, rapidly growing, lethal, dedifferentiated type responsible for 1.3–9.8% of all TCs, depending on geographical location [1,2,3]. ATC has a universally poor prognosis; however, there is insufficient up-to-date epidemiological data [4,5]. The traditional median survival is about five months, with an annual overall survival of 20% [6,7]. This grim prognosis demands proper counseling and defining goals of care with patients.

Tissue diagnosis of ATC can be difficult, especially for metastasis. This is because the immunohistochemistry staining for typical thyroid markers can be negative, and the ATC has many types and mixed patterns: three main histological growth patterns (spindle cell, pleomorphic giant cell, and squamoid) and two rare histological variants: (pauci-cellular and rhabdoid). Nevertheless, all ATCs are classified by the TNM system of the American Joint Committee on Cancer as stage IV A (localized intrathyroidal), B (regional extension (extrathyroidal) including lymph nodes), or C (metastatic). There is a high rate of metastasis to regional lymph nodes and distant organs, around 50%, at the time of diagnosis [7]. Here, we report a case of ATC with local pressure symptoms and gastric bleeding secondary to gastric metastases to highlight the challenges in its diagnosis and management.

## 2. Case Presentation

A 74-year-old Arab male patient was admitted with eight weeks’ symptomatic anemia for investigation. The patient presented with exertional dyspnea, generalized fatigue, melena, and a large right-side neck mass with a gradual and progressive pressure symptoms. The patient had a large thyroid mass with retrosternal extension. The right side was more prominent than the left, with tracheal deviation. The abdomen examination revealed an old surgical scar and no palpable masses. The rectal examination was positive for melena. The patient’s hemoglobin dropped from 14 g/dL to 7.9 g/dL over eight weeks. He underwent chest X-rays (Figure 1) that showed nodules in the left and right lower zones and a widening of the mediastinum (underlying mass or retrosternal goiter).

A computed tomography (CT) scan of the neck and chest showed a largely enhanced patchy soft tissue mass-like lesion arising from the right lobe of the thyroid (10 cm by 8 cm) extending upward into the soft tissue of the right side of the neck region almost to the level of the cricoid cartilage compressing and displacing to the left the upper two-thirds of the trachea. The lesion extended downward into the superior mediastinum between the main branches of the arch of the aorta. There were multiple enlarged mediastinal lymph nodes. Both lung fields showed widespread nodules of different sizes and shapes.

Abdominal CT revealed a large pedunculated nonobstructive mass in the greater curvature of the stomach, filling almost the whole gastric cavity but sparing the antrum and the pyloric canal. No evidence of a focal lesion was seen in the liver, and the right kidney was atrophied. There was no retroperitoneal lymphadenopathy; and the left kidney was normal (Figure 2).

The upper endoscopy showed a gastric mass that was biopsied along with a necrotic bleeding lesion (Figure 3). The stomach lesion biopsy showed an atypical spindle cell neoplasm. The specimen’s superficial and disrupted nature and poor preservation precluded accurate assessment and made interpretation difficult. However, in better-preserved tumor tissue, the overall morphological and immunohistochemical appearances, in particular SMA positivity, raised the possibility of a smooth muscle neoplasm with atypical changes. This limited material was insufficient to accurately determine the clinical behavior of this tumor.

The surgical management plan was discussed with the patient and family due to the complexity of the case and the need to manage both lesions simultaneously without definite pathologies, which is very critical. The gastric lesion was symptomatic, causing bleeding with significant drop in the hemoglobin, and required surgical excision. The clinical differential diagnosis was gastrointestinal stromal tumor (GIST), or leiomyoma, based on the CT scan, endoscopy, and gastric biopsy results. The thyroid mass with pressure symptoms extended into the retrosternal region suggested a malignant nature and also necessitated excision. The multidisciplinary teams (MDTs) recommended right-side hemithyroidectomy because of the lack of definite histopathology from the thyroid. On this basis, we reached a consensus with the patient and his family for a combined operation, starting with hemithyroidectomy and followed by excision of the gastric tumor. Prior Fine Needle Aspiration (FNA) and core biopsy from the thyroid were also inconclusive, showing only necrotic and non-viable tissue (the biopsy showed necrotic tissue with no evidence of malignancy; therefore, staged operation was primarily performed to diagnose, stage, or relieve patient pressure symptom).

The patient underwent a right hemithyroidectomy with a mini sternotomy to mobilize the retrosternal goiter. The gastric lesion was managed through midline laparotomy and wedge excision of the posterior wall of the stomach along the greater curvature with primary closure. The margins were negative (R0). The total amount of blood loss was 250 mL, and the patient was extubated and taken to the surgical intensive care unit for monitoring. The two surgeries were performed on the same day and by the same team and lasted for 3.5 h. He was discharged home after one week. Follow-up with oncology and surgery was arranged after one week, and a PET-CT scan was arranged for further staging.

The right thyroid specimen was almost entirely composed of a tan, gray tumor mass, measuring 13 cm in maximum dimension, whereas the tumor on the posterior wall of the stomach consisted of multiple fragments of tissue measuring 10 × 6 × 2 cm in aggregate. Histological examination showed the two tumors to be morphologically similar to each other in many areas; therefore, it indicates that one of the tumor sites was a metastasis from the other. It was therefore vital to determine the primary site due to treatment implications. It was apparent that the thyroid tumor was an anaplastic thyroid carcinoma, with a predominant sarcomatoid/spindle cell appearance and focal intimate association with an epithelial component that was arranged in a microfollicular pattern (Figure 4 and Figure 5). Angioinvasion of the spindle cell component was noted at the periphery of the mass (Figure 6).

The epithelial component was positive for thyroid markers PAX8 and thyroglobulin (Figure 7), whereas the spindle cell/sarcomatoid component only showed non-specific SMA, vimentin and calponin positivity (Figure 8 and Figure 9). A wide panel of antibodies, including keratins (Cam5.2, CKAE1/AE3, CK5/6), thyroid markers (PAX8, thyroglobulin, TTF1), caldesmon, P63, P40, GIST markers (CD117, DOG1, CD34), CD31 and desmin were negative in the sarcomatoid areas. Incidentally, a microscopic focus of well differentiated thyroid carcinoma was identified in one of the lymph nodes, revealing features similar to the epithelial component of the thyroid tumor (Figure 10). Since the entire right hemithyroidectomy specimen was almost exclusively composed of tumor tissue and the left thyroid lobe was not excised, background non-tumor thyroid parenchyma could not be assessed.

It is well known that the spindle cell/sarcomatoid component of anaplastic carcinoma is frequently non-reactive or weakly positive for cytokeratins as well as with numerous other markers of epithelial and thyroid differentiation, such as TTF1, PAX8, thyroglobulin, EMA, and CEA, as noted in this case. Since most anaplastic thyroid carcinomas are believed to arise from dedifferentiation of a well differentiated or poorly differentiated thyroid carcinoma, the identification of a minor follicular epithelial component that is intimately associated with the sarcomatoid component is crucial in the diagnosis of this case. This is further strengthened by the presence of a small focus of metastatic well differentiated thyroid carcinoma in a lymph node that bears similar architectural and cytological features to the epithelial component of the main thyroid tumor.

Diagnosing anaplastic thyroid carcinoma can be challenging as the markers of epithelial and thyroid differentiation are frequently lost, especially in the sarcomatoid variants. Other tumors that may show similar morphology such as poorly differentiated thyroid carcinoma, papillary thyroid carcinoma with spindle cell or squamous metaplasia, papillary thyroid carcinoma with fasciitis-like stroma, malignant fibrous histiocytoma, metastatic poorly differentiated or undifferentiated carcinoma, melanoma, true sarcomas and lymphoma were all considered in the differential diagnoses and excluded on clinical and immunohistochemical grounds.

Histological examination of the gastric mass, on the other hand, showed involvement of the gastric wall by a malignant spindle cell/sarcomatoid tumor, extending into the surface mucosa (Figure 11). This tumor was similar histologically and immunohistochemically to the sarcomatoid component of the anaplastic thyroid carcinoma (Figure 12). The gastric tumor was positive for SMA (Figure 13) and calponin and negative for all other markers that were also performed and found to be negative in the sarcomatoid component of the thyroid mass.

Since the thyroid mass was proven to be a primary spindle cell/sarcomatoid type of anaplastic thyroid carcinoma with lymph node metastasis of the epithelial component further confirming the diagnosis, the gastric mass was therefore considered to be metastasis of the spindle cell/sarcomatoid component of the anaplastic thyroid carcinoma. The presence of angioinvasion of only the spindle cell component of the thyroid tumor is the most likely explanation for the gastric metastasis displaying the spindle cell features exclusively. No mutation was detected for cKIT, PDGFRA, and BRAF. The cKIT and PDGFRA mutation screening was carried out as a high-grade gastrointestinal stromal tumor (GIST) was considered initially amongst the differential diagnoses, and any activating mutations of cKIT or PDGFRA would have been helpful in predicting imatinib response. These were tested after genomic DNA was extracted from formalin-fixed paraffin-embedded tissue (FFPE) and analyzed by using Sanger sequencing of exon 9, 11, 13 and 17 of the cKIT gene and exon 12 and 18 of PDGFRA gene. V600E in exon 16 of BRAF gene was tested by real-time PCR assay.

PET-CT scans 2 weeks after the surgery showed a right-sided cervical mass likely involving lymph nodes in the direction of the thoracic inlet. There are bilateral multiple lung, bone, and liver metastases. There were subcutaneous metastases: one in the scalp (Figure 14) in the left parietal region and one in the intergluteal region. One month after the primary surgery, radiotherapy started on the femur and neck (30 Gy in 10 fractions (3.0 Gy/fraction) to the femur for palliative course and 40 Gy in 15 fractions (2.66 Gy/fraction) to the neck. Two months later, two cycles of chemotherapy were administrated (the first cycle was a combination of Paclitaxel and carboplatin while the second cycle one week later included only Paclitaxel). As no mutation was detected in the final biopsy, target therapy was not indicated. The patient developed septic shock secondary to bilateral pulmonary infiltration and died four and half months after the primary surgery.

## 3. Discussion

Anaplastic thyroid carcinoma is an aggressive, lethal, rare subtype of thyroid cancer with a universally poor prognosis. Most ATC patients die early after diagnosis [3–9 months] regardless of treatment modalities, and 20% survive the first year [6,7]. It tends to affect older patients with poorer prognosis compared to younger patients, mainly if metastasis is present.

Anaplastic thyroid carcinoma has three main histological growth patterns and two rare histological variants: spindle cell, pleomorphic giant cell, and squamoid; often, mixed patterns are found. The rare variants are pauci-cellular and rhabdoid [6].

Multimodality treatment is the recommended management regime for ATC; despite overall poor prognosis and known chemo/radio-resistance, some patients, particularly younger patients, reveal that there is more to explore and learn about this tumor and its behavior and prognosis [7,8,9,10].

So far, no unified ATC treatment for improving overall survival has been established. Several guidelines discuss treatment options, including surgery, radio- and/or chemotherapy. Recently, the efficacy of targeted therapy approaches has been reported in case reports. Moreover, checkpoint inhibitors represent new treatment options [11]. In our case, immunotherapy was not administered. The United States Food and Drug Administration (FDA) approved the use of a combination of the BRAF inhibitor dabrafenib with the mitogen-activated protein kinase for the treatment of locally advanced or metastatic BRAF V600E-mutant ATC. Although selective targeting therapy for a single mutation may work, there is a risk of secondary failure [12]. In our case, BRAF V600E mutation was not detected. Although BRAF V600E mutations are common in papillary thyroid carcinomas, the reported incidence in anaplastic thyroid carcinomas ranges from 10% to 45%. Other common mutations known to be associated with anaplastic thyroid carcinoma include TERT (usually co-occurring with BRAF V600E mutation), TP53 (in up to 70% cases) and PIK3CA (prevalence ranges from 5 to 18 for TERT, TP53 and PIK3CA were not carried out in our case unfortunately, and we acknowledge this limitation). However, while the presence or absence of these mutations reflects the molecular genetics and pathogenesis of this tumor, thereby playing a role in patient management and treatment, their detection is not an essential component for tumor diagnosis. In addition, we were unable to test BRAF V600E mutation in the lymph node metastasis due to the very small tumor focus, making it unsuitable for molecular testing.

Metastasis in the stomach is rare, reported in 4% of 45 autopsy cases from a single institute [13]. A few cases were reported in the literature with ATC with stomach metastasis; one had an asymptomatic mass (localized gastric wall thickness) on follow-up CT confirmed with upper endoscopy and biopsy, while another one presented with gastrointestinal bleeding like the current case [14,15,16,17]. The first case had no gastric surgery and rapidly deteriorated and died. The second one was treated by hemithyroidectomy, radical neck dissection and radioactive therapy. Nine months later, he developed metastasis to the brain, axilla and stomach. No gastric surgery was performed but biopsy was, and the outcome was not reported.

## 4. Conclusions

This is a rare case of metastatic ATC with GI bleeding, challenging diagnosis, and short survival. ATC should be suspected in metastatic thyroid cancer even when a pattern of presentation is atypical. Surgery remains the appropriate palliative modality. This disease’s pathology, behavior, and targeted new treatment modalities must be explored further.

## Figures and Tables

**Figure 1:**
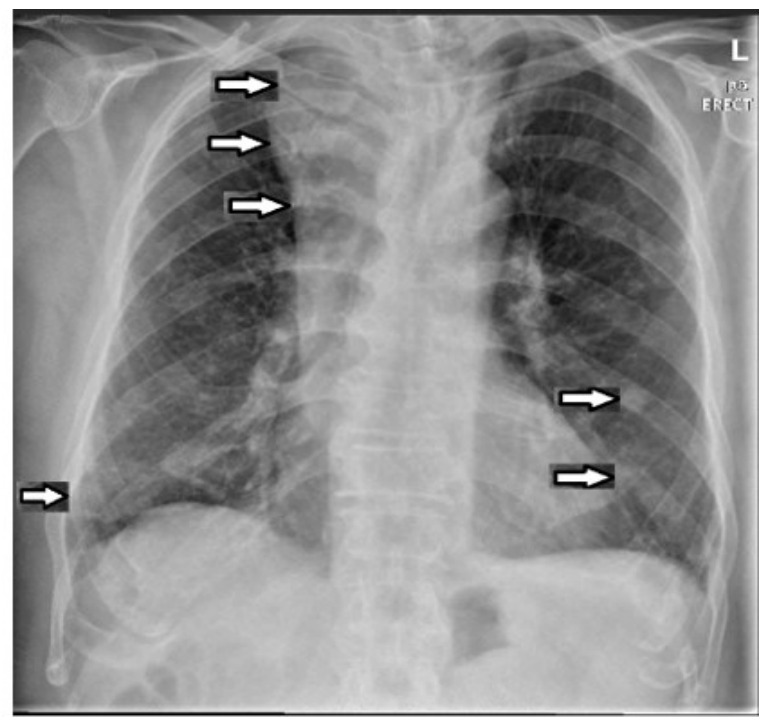
Chest x-ray showed a nodule in the left and right lower zones (lower arrows), a widening of the mediastinum quire, and an underlying mass or retrosternal goiter (upper arrows).

**Figure 2:**
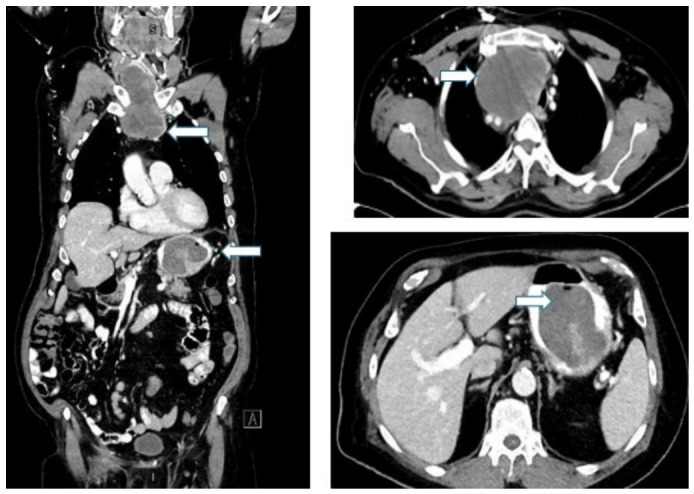
CT scans revealed a large mass in the greater curvature of the stomach (arrows on the coronal and cross-sectional views) and a thyroid mass extended to the mediastinum (arrow on the upper part of the coronal view).

**Figure 3:**
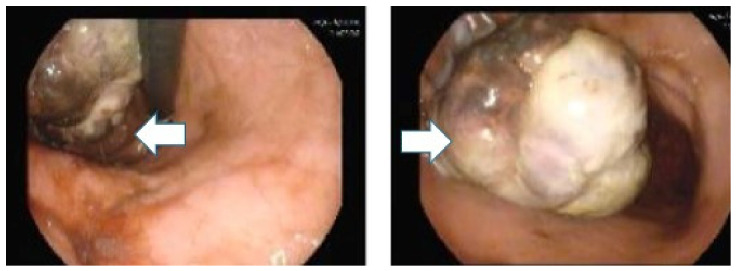
Stomach mass showed friable masses.

**Figure 4:**
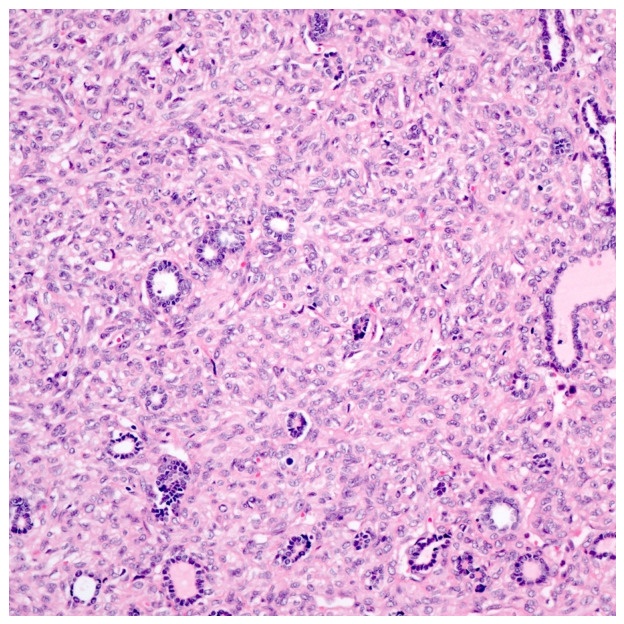
Anaplastic thyroid carcinoma, composed of a predominant spindle cell/sarcomatoid morphology, associated with a small epithelial component composed of microfollicles (H and E ×10).

**Figure 5:**
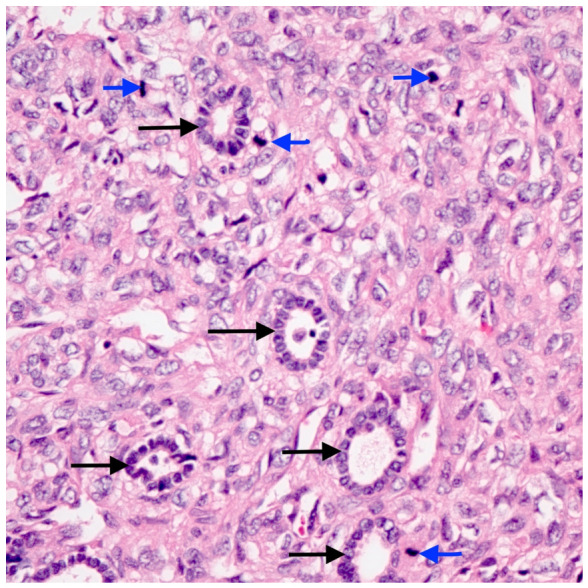
Microfollicles (black arrows) are composed of cells with vesicular nuclei with little nuclear overlap. The spindle cell component displays marked nuclear pleomorphism and is intimately associated with the epithelial component. Mitoses (blue arrows) are easily discernible (H and E ×40).

**Figure 6:**
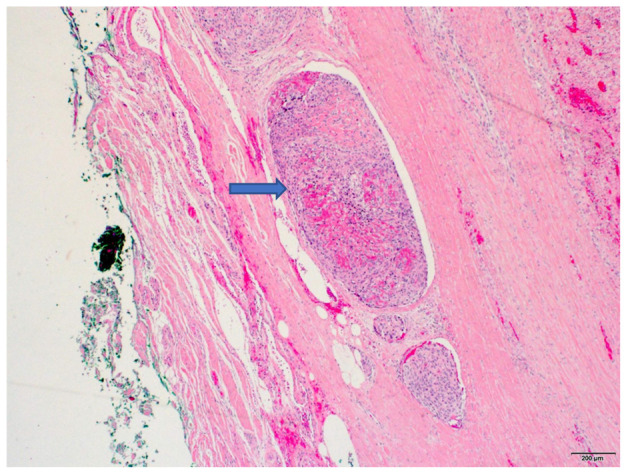
Angioinvasion of the spindle cell component (blue arrow) was noted at the edge of the thyroid tumor (H and E ×4).

**Figure 7:**
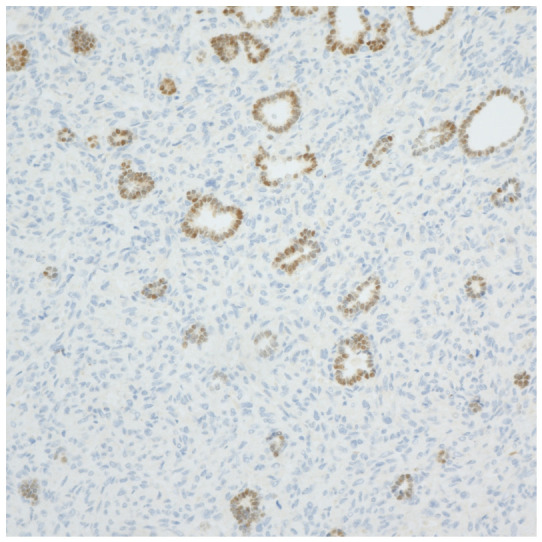
PAX8 highlights the epithelial component. Note the background negative staining in the spindle cell component (×20).

**Figure 8:**
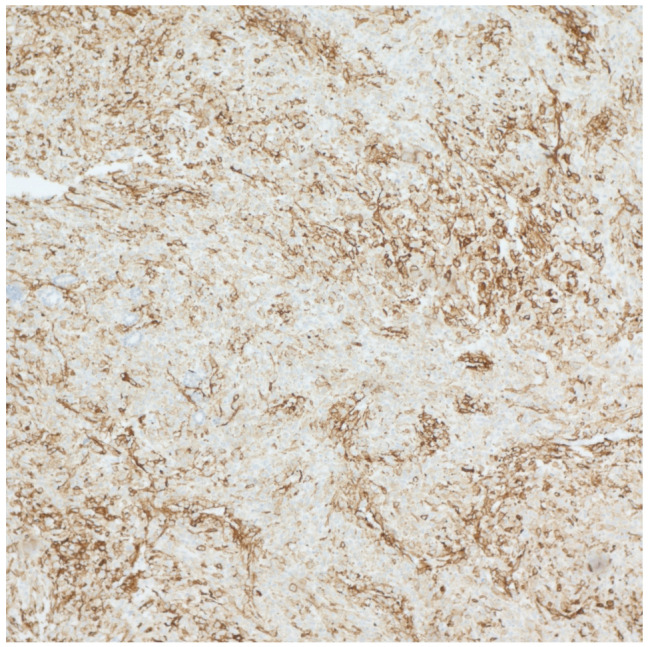
The spindle cell component shows variable but strong SMA positivity (×10).

**Figure 9:**
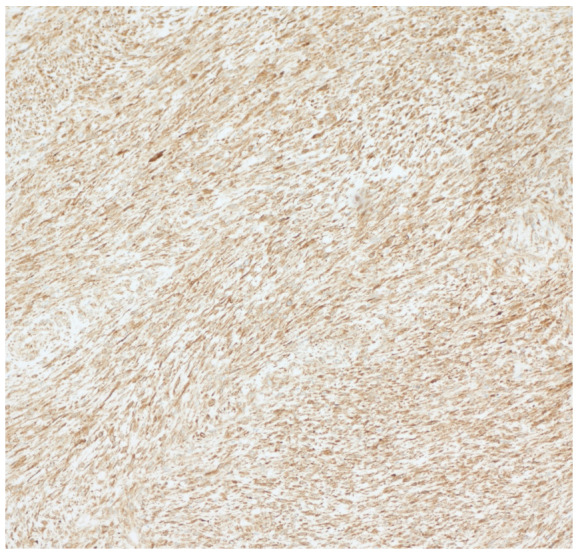
Diffuse strong calponin positivity is also noted in the spindle cell component (×10).

**Figure 10:**
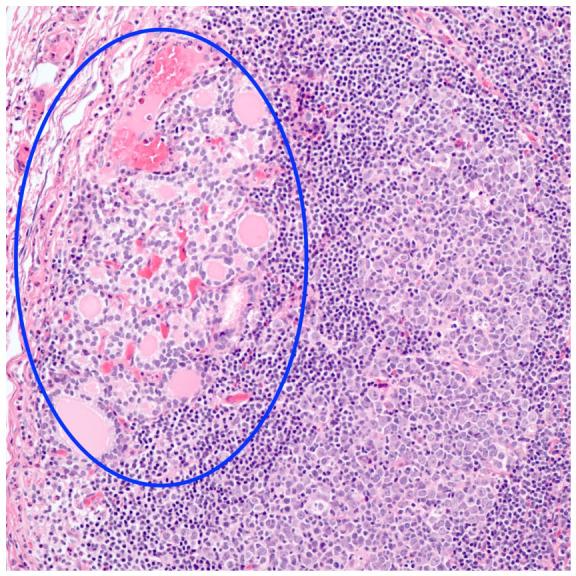
A small focus of metastatic thyroid carcinoma (blue circle), composed of well differentiated microfollicles, similar to that seen in the epithelial component of the anaplastic thyroid carcinoma (H and E ×40).

**Figure 11:**
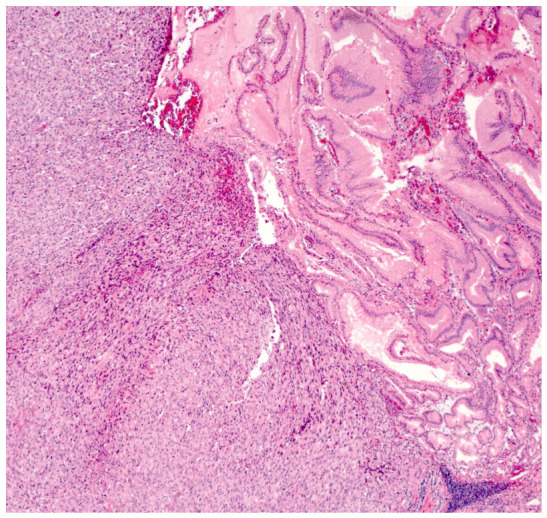
Gastric wall metastasis of anaplastic thyroid carcinoma, composed exclusively of the spindle cell component. Note the benign gastric mucosa on the right of the image (H and E ×4).

**Figure 12:**
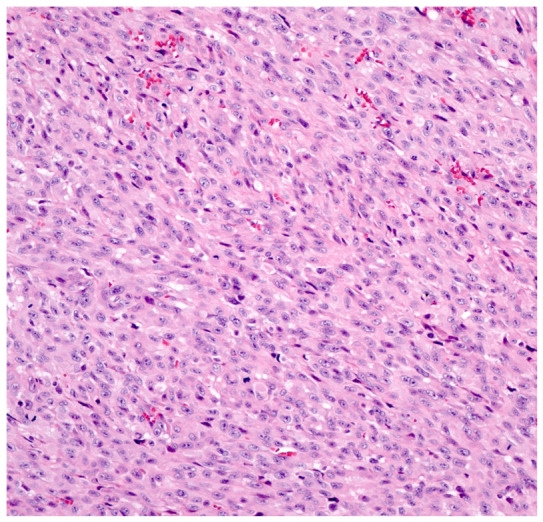
Higher-power view of the gastric metastasis showing spindle cell/sarcomatoid component with frequent mitoses. No epithelial component is seen (H and E ×20).

**Figure 13:**
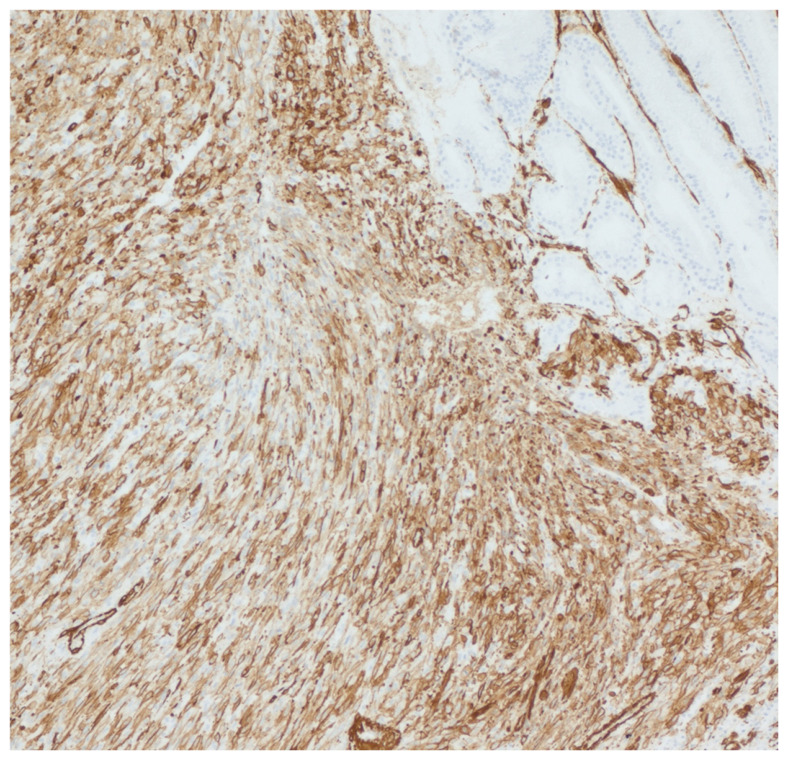
The metastatic tumor shows widespread SMA positivity, similar to the spindle cell component of the anaplastic thyroid carcinoma (×10).

**Figure 14:**
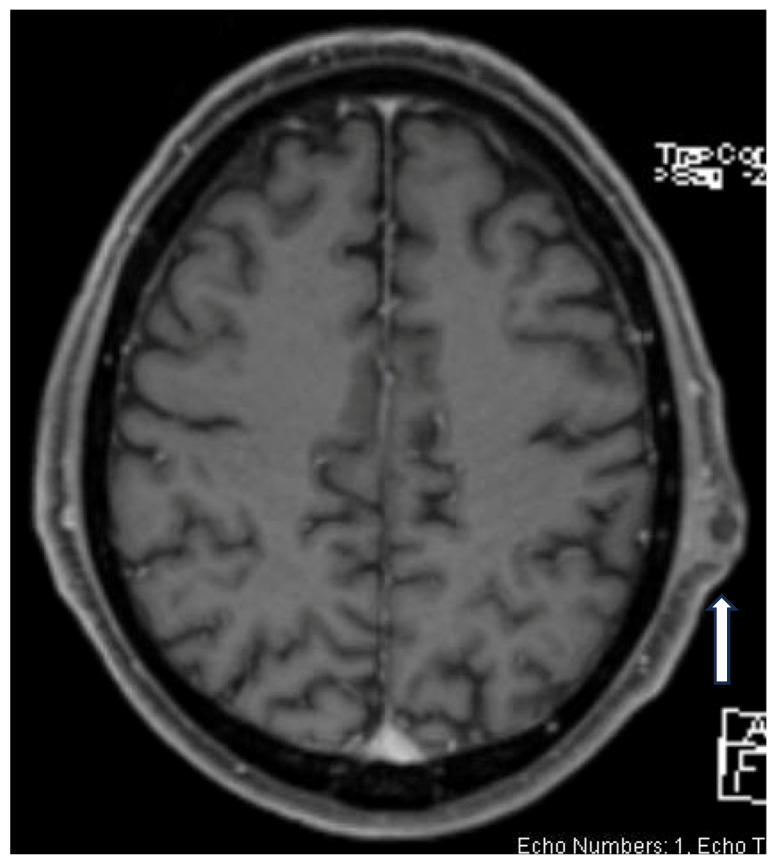
Subcutaneous metastases (arrow) on the left side of the scalp (parietal region).

## Data Availability

Not applicable as no data was generated.

## Abbreviations

ATC	Anaplastic thyroid carcinoma
MDT	Multidisciplinary team
GIST	Gastrointestinal stromal tumor
CT	Computerized tomography
PET	Positron Emission Tomography
